# Spike propagation through the dorsal root ganglia in an unmyelinated sensory neuron: a modeling study

**DOI:** 10.1152/jn.00226.2015

**Published:** 2015-09-02

**Authors:** Danielle Sundt, Nikita Gamper, David B. Jaffe

**Affiliations:** ^1^Department of Biology, UTSA Neurosciences Institute, University of Texas at San Antonio, San Antonio, Texas;; ^2^Department of Pharmacology, Hebei Medical University, Shijiazhuang, People's Republic of China; and; ^3^Faculty of Biological Sciences, School of Biomedical Sciences, University of Leeds, Leeds, United Kingdom

**Keywords:** nociceptor, DRG, computer model, unmyelinated axon, action potential, KCNQ

## Abstract

Unmyelinated C-fibers are a major type of sensory neurons conveying pain information. Action potential conduction is regulated by the bifurcation (T-junction) of sensory neuron axons within the dorsal root ganglia (DRG). Understanding how C-fiber signaling is influenced by the morphology of the T-junction and the local expression of ion channels is important for understanding pain signaling. In this study we used biophysical computer modeling to investigate the influence of axon morphology within the DRG and various membrane conductances on the reliability of spike propagation. As expected, calculated input impedance and the amplitude of propagating action potentials were both lowest at the T-junction. Propagation reliability for single spikes was highly sensitive to the diameter of the stem axon and the density of voltage-gated Na^+^ channels. A model containing only fast voltage-gated Na^+^ and delayed-rectifier K^+^ channels conducted trains of spikes up to frequencies of 110 Hz. The addition of slowly activating KCNQ channels (i.e., K_V_7 or M-channels) to the model reduced the following frequency to 30 Hz. Hyperpolarization produced by addition of a much slower conductance, such as a Ca^2+^-dependent K^+^ current, was needed to reduce the following frequency to 6 Hz. Attenuation of driving force due to ion accumulation or hyperpolarization produced by a Na^+^-K^+^ pump had no effect on following frequency but could influence the reliability of spike propagation mutually with the voltage shift generated by a Ca^2+^-dependent K^+^ current. These simulations suggest how specific ion channels within the DRG may contribute toward therapeutic treatments for chronic pain.

sensory information from the periphery, including pain, must pass through the dorsal root ganglion (DRG) before reaching the spinal cord. Several lines of evidence support the hypothesis that the DRG “gates” nociceptive signaling. First, analgesia arises from hyperpolarizing the resting potential of rodent nociceptive DRG neurons (Du et al. 2014). Pharmacological blockade or the activation of ion channels that induce hyperpolarization, limited to the DRG, reduces nocifensive behavior. Second, electrical stimulation of the DRG in humans reversibly provides pain relief (Deer et al. 2012; Liem et al. 2013; Pope et al. 2013). Although it is not clear how direct electrical stimulation of the DRG transiently limits pain signaling but does not cause pain itself, this suggests a mechanism where action potential propagation through the DRG is reduced. Third, action potentials are low-pass filtered by the DRG in rodent nociceptive neurons (Gemes et al. 2012; Luscher et al. 1994b).

The pseudo-unipolar morphology of sensory neurons within the DRG, where the peripheral axon of a sensory neuron bifurcates into the axon continuing on to the spinal cord (the central branch) and the stem axon that joins to the soma (Hendry et al. 1999), has long suggested the innate possibility for low-pass filtering (Zhou and Chiu 2001). By means of impedance mismatch, the safety factor for spike propagation may be reduced (Bucher and Goaillard 2011; Debanne et al. 2011; Goldstein and Rall 1974; Manor et al. 1991; Sasaki 2013), and as a result, variation of membrane potential and membrane conductance in the vicinity of the T-junction could potentially regulate sensory information reaching the spinal cord. There is substantial experimental evidence from both myelinated and unmyelinated sensory neurons that spikes indeed fail at some point during their passage through DRG, most likely at the T-junction (Djouhri et al. 2001; Du et al. 2014; Fang et al. 2005; Gemes et al. 2012; Luscher et al. 1994b; Stoney 1985, 1990).

Although the impedance mismatch at the T-junction suggests a mechanism for reducing the safety factor for action potential conduction through the DRG, morphological studies of axonal diameter suggest that orthodromic spike propagation through the DRG should be quite favorable. The diameter of the unmyelinated central axon is 50% smaller than the diameter of the peripheral axon (Ha 1970; Hoheisel and Mense 1986; Suh et al. 1984; Zhang et al. 1998). This is consistent with measurements of conduction velocity, which is generally faster in the peripheral axon compared with the central branch for both myelinated and unmyelinated sensory neurons (Czeh et al. 1977; Obreja et al. 2010; Raymond et al. 1990; Suh et al. 1984; Tigerholm et al. 2014; Waddell et al. 1989).

Unmyelinated C-fibers are a major type of DRG sensory neuron conveying pain information, in addition to mechanical, chemical, and thermal stimuli (Dubin and Patapoutian 2010). They usually fire at rates <20 Hz (Chen and Levine 2003; Leem et al. 1993; Long 1977; Yeomans et al. 1996; Yeomans and Proudfit 1996) but may approach 100 Hz (Chen and Levine 2003; Kress et al. 1992; Leem et al. 1993; Yeomans et al. 1996; Yeomans and Proudfit 1996), and high-frequency firing is believed to be important in the development of chronic pain (Fang et al. 2002). Understanding how C-fiber signaling is influenced by the morphology of the T-junction and the local expression of ion channels regulating action potential conduction is important not only for understanding normal pain signaling but also for developing clinical approaches that might restore proper filtering of signals through the DRG in patients with chronic pain.

In this study, we sought to understand how the morphological features of axons proximal to the T-junction of unmyelinated nociceptors (C-fibers) influence the reliability of spike propagation. It is speculated that other cellular mechanisms near the site of bifurcation, namely, those leading to hyperpolarization (Du et al. 2014), may work in conjuncture with whatever impedance mismatch the T-junction provides to successfully limit high-frequency signals. We used computational modeling to investigate the interaction of axonal morphology, somatic and axonal excitability, and slowly activating membrane conductances on spike conduction through the DRG. We found that *1*) the impedance mismatch from the T-junction alone does not account for adequate filtration of orthodromic signals and *2*) slowly activating voltage-dependent K^+^ potassium currents (M-channels) may contribute in part to low-pass filtering, but *3*) a slower, hyperpolarizing conductance [such as a small-conductance (SK)-like Ca^2+^-dependent K^+^ channel] is required to reduce the following frequency to physiological levels.

## MATERIALS AND METHODS

### Simulation and Analysis

Simulations were performed using NEURON (Hines and Carnevale 1997, 2000) on an Intel-based Macintosh computer. Simulation results were analyzed using IgorPro analysis software (Wavemetrics, Lake Oswego, OR).

### Morphology

**Fig. 1.**
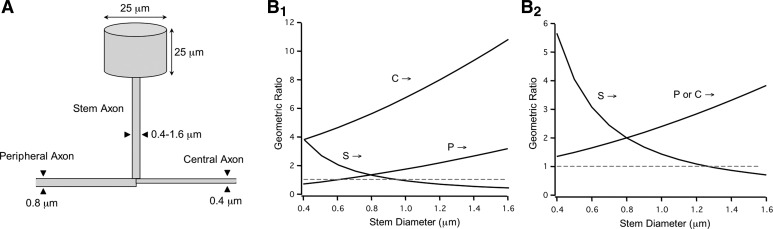
Range of geometric ratios at the T-junction. *A*: diagram illustrating the exemplar geometry for the sensory neuron model. *B*: geometric ratios calculated over a range of axon diameters for spikes originating from the stem axon (S→), central axon (C→), and peripheral axon (P→). Dashed line represents unity. *B*_1_ and *B*_2_ present the geometric ratios calculated from model with a 0.4- and 0.8-μm-diameter central axon, respectively.

The soma was modeled as a cylinder with a diameter and length of 25 μm (Hayar et al. 2008; Rose et al. 2013; Zhang et al. 1998) and therefore a capacitance of 20 pF [when isolated and assuming membrane capacitance (*C*_m_) of 1 μF/cm^2^]. The diameter of the peripheral axon was 0.8 μm, and in most simulations, the diameter of the central axon was 0.4 μm (Ha 1970; Hoheisel and Mense 1986; Suh et al. 1984; Zhang et al. 1998). A stem axon connects the soma to where the peripheral and central axons join, generally referred to as the T-junction (Fig. 1*A*). Peripheral and central axon segments 100 μm proximal to the T-junction were subdivided into 100 compartments. The stem axon (also subdivided into 100 compartments) in most simulations had a length of 150 μm and a diameter of 1.4 μm, unless otherwise noted. Both the peripheral and central axons were extended an additional 5 mm and further subdivided into 100 compartments. Increasing the number of sections had no effect on the results. Simulations were performed with time steps of 1–25 ms.

### Passive Properties

Specific membrane resistivity (*R*_m_) for all compartments was 10,000 Ω·cm^2^ (Choi and Waxman 2011; Neishabouri and Faisal 2014), and axial resistance (*R*_a_) was 100 Ω·cm (Choi and Waxman 2011; Luscher et al. 1994b). The slowest time constant (τ_o_) of the passive somatic response to a current step was 8 ms, and somatic input resistance (*R*_N_) was 274 MΩ (Hayar et al. 2008; Zhang et al. 1998), giving the soma an apparent capacitance (τ_o_/*R*_N_) of 29 pF (Du et al. 2014; Gao et al. 2012).

### Active Conductances

Voltage-gated Na^+^ (Na_V_) and delayed-rectifier K^+^ channels (K_DR_) were expressed in all compartments with a density of 40 mS/cm^2^, except at the soma, where Na^+^ conductance (*G*_Na_) was halved to 20 mS/cm^2^ (Matsumoto and Rosenbluth 1985) to achieve an action potential amplitude of 73 mV at the soma (Djouhri and Lawson 2001). The voltage dependence of Na_V_ was adjusted to be approximately midway between values reported for Na_V_1.7 and Na_V_1.8 channels expressed in DRG nociceptive neurons (Cummins and Waxman 1997; Sheets et al. 2007). KCNQ channels (K_V_7) underlying a slowly activating voltage-dependent K^+^ current (K_KCNQ_) were added to the model where noted (Yamada et al. 1988). In some simulations, high-threshold L-type voltage-gated Ca^2+^ channels (Ca_V_) (Jaffe et al. 1994) and SK Ca^2+^-dependent K^+^ channels (K_SK_) (Aradi and Holmes 1999) were also added. Equations for voltage- and Ca^2+^-dependent conductances are listed below.

#### Na_V_ current.

Equations for Na_V_ current conductance are adapted from Cummins et al. (2007), Sheets et al. (2007), and Traub et al. (1991):

$$\begin{array}{cc} I_{Na}={\overset{¯}{g}}_{Na}\cdot m^{3}h\cdot \left(V-E_{Na}\right) & \\ m_{\infty }\left(V\right)=\frac{\alpha_{m}\left(V\right)}{\alpha_{m}\left(V\right)+\beta_{m}\left(V\right)} & h_{\infty }\left(V\right)=\frac{\alpha_{h}\left(V\right)}{\alpha_{h}\left(V\right)+\beta_{h}\left(V\right)}\\ \tau_{m}\left(V\right)=\frac{1}{\alpha_{m}\left(V\right)+\beta_{m}\left(V\right)} & \tau_{h}\left(V\right)=\frac{1}{\alpha_{h}\left(V\right)+\beta_{h}\left(V\right)}\\ \alpha_{m}\left(V\right)=0.55\left(7.1-V\right)/\exp\left(\frac{7.1-V}{4}\right) & \beta_{m}\left(V\right)=\frac{0.48\left(V-46.1\right)}{\exp\left(\frac{V-46.1}{5}\right)-1}\\ \alpha_{h}\left(V\right)=0.22\exp\left(\frac{23-V}{18}\right) & \beta_{h}\left(V\right)=\frac{6.92}{1+\exp\left(\frac{46-V}{5}\right)} \end{array}$$

#### K_DR_ current.

Equations for K_DR_ current conductance are adapted from Borg-Graham (1987):

$$\begin{array}{cc} I_{K(DR)}={\overset{¯}{g}}_{K(DR)}\cdot n^{3}l\cdot \left(V-E_{K}\right) & \\ \alpha_{n}\left(V\right)=\frac{\exp\left(-5\times 10^{-3}\cdot \left(V+32\right)\cdot 9.648\times 10^{4}\right)}{2562.35} & \beta_{n}\left(V\right)=\frac{\exp\left(-2\times 10^{-3}\cdot \left(V+32\right)\cdot 9.648\times 10^{4}\right)}{2562.35}\\ \alpha_{l}\left(V\right)=\frac{\exp\left(2\times 10^{-3}\cdot \left(V+61\right)\cdot 9.648\times 10^{4}\right)}{2562.35} & \beta_{l}\left(V\right)=\frac{\exp\left(-2\times 10^{-3}\cdot \left(V+32\right)\cdot 9.648\times 10^{4}\right)}{2562.35} \end{array}$$

#### K_KCNQ_ current.

Equations for K_KCNQ_ current conductance are adapted from Yamada et al. (1988):

$$\begin{array}{cc} I_{K(M)}={\overset{¯}{g}}_{K(M)}\cdot m\cdot \left(V-E_{K}\right) & \\ m_{\infty }\left(V\right)=1/(1+\exp\left(-\left(V+35\right)/10\right))\;\; & \tau_{m}\left(V\right)=\frac{1,000}{3.3\left\{\exp\left[\left(V+35/20\right)+\exp\left(-\left(V+35\right)/20\right)\right]\right\}/3.54} \end{array}$$

#### Ca_V_ current.

Equations for Ca_V_ current conductance are adapted from Migliore et al. (1995):

$$\begin{array}{cc} I_{Ca}={\overset{¯}{g}}_{Ca}\cdot m^{2}\cdot \left(V-E_{Ca}\right) & E_{Ca}=\ln\left(\frac{2}{{\left[Ca\right]}_{i}}\right)\frac{3.08\times 10^{5}R}{2F}\\ \alpha_{m}\left(V\right)=\frac{15.69\cdot \left(81.5-V\right)}{\exp\left[\left(81.5-V\right)/10\right]-1} & \beta_{m}\left(V\right)=0.29\exp\left(-V/10.86\right) \end{array}$$

where *R* is the gas constant and *F* is the Faraday constant.

#### Ca^2+^-dependent SK current and intracellular Ca^2+^ dynamics.

The following equations are adapted from Aradi and Holmes (1999):

$$\begin{array}{cc} I_{K\left(Ca\right)}={\overset{¯}{g}}_{K(Ca)}\cdot q^{2}\cdot \left(V-E_{K}\right) & \\ \alpha_{q}\left({\left[Ca\right]}_{i}\right)=\frac{0.00246}{\exp\left[\left(12\cdot \log\left({\left[Ca\right]}_{i}^{3}\right)+28.48\right)/-4\right]} & \beta_{q}\left({\left[Ca\right]}_{i}\right)=\frac{0.006}{\exp\left\{\left[12\cdot \log\left({\left[Ca\right]}_{i}^{3}\right)+60.4\right]/35\right\}}\\ \frac{d{\left[Ca\right]}_{i}}{dt}=-0.026\cdot I_{Ca}-\frac{\left({\left[Ca\right]}_{i}-{\left[Ca\right]}_{rest}\right)}{20} & \end{array}$$

The equilibrium potentials for Na^+^ (*E*_Na_) and K^+^ (*E*_K_) were +55 and −90 mV, respectively (Czeh et al. 1977; Keynes and Ritchie 1965; Villiere and McLachlan 1996). NEURON's mechanism for calculating intracellular and extracellular Na^+^ and K^+^ dynamics was applied to influence *E*_Na_ and *E*_K_ and was maintained with an electrogenic Na^+^-K^+^ pump where noted (Canavier 1999; Fleidervish et al. 2010). The leak equilibrium potential (*E*_leak_) was calculated from the sum of resting voltage- and Ca^2+^-dependent currents to achieve a resting potential (*E*_rest_) of −60 mV (Du et al. 2014; Gemes et al. 2012; Hayar et al. 2008).

Action potentials were initiated in either the peripheral or central axons 4.6 mm from the T-junction by depolarizing current steps (0.2 nA, 1-ms duration). The peripheral axon (diameter 0.8 μm) had a conduction velocity (CV) of 0.54 m/s, whereas the central axon (diameter 0.4 μm) had a CV of 0.28 m/s (Ha 1970; Hoheisel and Mense 1986; Suh et al. 1984; Zhang et al. 1998).

## RESULTS

### Validation of the Model

The model recapitulated three important physiological features of unmyelinated DRG sensory neurons. First, the geometry and incorporated cellular properties resulted in a model with a somatic input resistance (see materials and methods) within 5% of values reported by Hayar et al. (2008) and 20% of those reported by Zhang et al. (1998) using whole cell patch-clamp recordings in acute DRG preparations. The latter value could be better matched with the use of a greater value for *R*_m_, without altering the general features of the model. Second, the amplitude and waveform of the somatic action potential were similar to those again measured using whole cell recording (Gamper N, unpublished data; Hayar et al. 2008; Zhang et al. 1998). Third, the CV of action potentials in the peripheral and central axons, 0.54 and 0.28 m/s, respectively, were comparable with published data (Ha 1970; Hoheisel and Mense 1986; Suh et al. 1984; Zhang et al. 1998).

### Morphological Influences on Spike Propagation

The likelihood that an action potential propagates through a branch point depends in part on the relative impedance of the parent axon with respect to the two daughter axons (Debanne et al. 2011; Goldstein and Rall 1974). The geometrical ratio (GR) of axonal diameter (diam), [(diam_daughter1_)^3/2^ + (diam_daughter2_)^3/2^]/(diam_parent_)^3/2^, provides a relative measure of this impedance mismatch. When this ratio is greater than unity, action potential waveforms will be filtered across the branch point. At a critical value of this ratio, spike propagation will fail. Ultrastructural and intracellular labeling studies suggest that within the DRG, the diameter of the central axon is significantly smaller than that of the peripheral axon (Ha 1970; Suh et al. 1984). To evaluate how stem axonal morphology at the T-junction influences action potential propagation, GR was calculated for measured values of peripheral and central axon diameters (0.8 and 0.4 μm, respectively) over a range of stem diameters (Fig. 1*B*_1_). GR for orthodromic propagation (i.e., spikes originating from the periphery) is greater than unity when stem axon diameter is >0.6 μm. If the T-junction affects orthodromic spikes, stem axon diameter should be the same as or larger than peripheral axon diameter. Assuming that the stem axon has a diameter of 1.2–1.4 μm (Luscher et al. 1994b), we find a GR of 2.3–2.6. In contrast, in the antidromic direction (i.e., spikes originating from the central axon) GR is >4 for stem diameters >0.4 μm. This suggests significant filtering of propagating signals, and the higher likelihood for spike failure, across a wide range of physiologically relevant diameters. Finally, for spikes originating from the soma (e.g., experimentally stimulated or ectopically generated action potentials), GR was at or below unity for stem diameters >1.0 μm. This indicates highly reliable propagation across a similar range of stem diameters.

For comparison, we examined the alternative condition where the diameter of the central axon is the same diameter as the peripheral axon (Fig. 1*B*_2_). In this case, the ratio for both orthodromic and antidromic propagation was the same across all diameters. In other words, the influence of the T-junction on orthodromic signal propagation should be the same as for antidromic propagation. Somatically generated signals, in contrast, are at or below unity for stem diameters 1.2 μm or greater. Taking these findings together, and assuming that *R*_a_ and *C*_m_ are the same across all of the axons, if the morphology of the T-junction significantly participates in filtering spike propagation, then the diameter of the stem axon needs to be greater than the diameter of the peripheral axon.

**Fig. 2.**
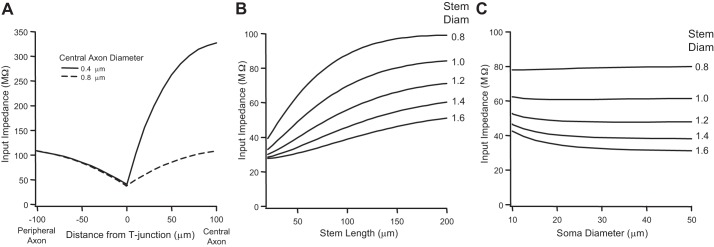
Axonal and somatic morphology effects on T-junction input impedance. *A*: spatial profile of input impedance ±100 μm on either side of the T-junction, calculated at a frequency of 250 Hz (stem axon length = 150 μm). Input impedance was calculated for central axon diameters of 0.4 (solid line) and 0.8 μm (dashed line). *B*: T-junction input impedance plotted as a function of stem axon length and diameter (central axon diameter 0.4 μm). *C*: T-junction input impedance plotted as a function of somatic diameter and stem axon diameter (central axon diameter 0.4 μm). Diam, diameter.

An impedance mismatch at the T-junction lowers the safety factor for action potential propagation by decreasing axial current and spike amplitude (Debanne 2004). At any particular location along an axon's length, input impedance (*Z*_N_) influences the amplitude of the spike (of course, along with the repertoire of voltage-gated conductances underlying the action potential). We calculated *Z*_N_ (for a frequency of 250 Hz based on an assumption of a spike half-width of 2 ms) as a function of distance along the peripheral and central axons on either side of the T-junction (Fig. 2*A*). *Z*_N_ decreases from 110 to 49 MΩ approaching the T-junction and rapidly rises to 325 MΩ on the opposing side. The asymmetry of *Z*_N_ around the T-junction is consistent with the calculated GRs for orthodromic and antidromic conduction (see Fig. 1*B*_1_). Again, for comparison, when the central axon diameter matches the diameter of the peripheral axon, the spatial profile *Z*_N_ around the T-junction is symmetrical. Similar peripheral/central axon *Z*_N_ profiles could also be achieved by branch-specific differences in *R*_a_ (not shown).

To better understand how stem axon morphology influences *Z*_N_ at the T-junction, we plotted *Z*_N_ at the T-junction as a function of stem axon length and diameter. As expected, *Z*_N_ (for 250-Hz signals, based on assumption of a spike half-width of 2 ms) at the T-junction increases with stem length and decreases with stem diameter (Fig. 2*B*). At the extreme of a large stem diameter and small stem length, where the stem axon is electronically short, we observe the lowest value for *Z*_N_ at the T-junction. Conversely, *Z*_N_ is greatest with an electrotonically long stem axon (small diameter and extensive length). Assuming the stem axon diameter is in the range of 1.2–1.4 μm (Ha 1970; Luscher et al. 1994a), variation in stem axon length between 50 and 200 μm results in an 82% and 85% difference in *Z*_N_, respectively. At stem lengths >200 μm, *Z*_N_ becomes relatively constant with increasing axon length. Significant variation in stem length and diameter could potentially result in heterogeneous differences in spike filtering at the T-junction.

Can somatic membrane influence *Z*_N_ at the T-junction? The diameters of putative C-fiber sensory neurons are generally smaller than for myelinated cells but may range between 10 and 50 μm (Hoheisel and Mense 1987; Rose et al. 2013; Zhang et al. 1998). Somatic diameter <20 μm only affected *Z*_N_ at the T-junction when the stem axon was electrotonically short (Fig. 2*C*), when the stem axon diameter was >1.2 μm or its length <150 μm. Otherwise, somatic diameter had no major influence on *Z*_N_ at the T-junction. In other words, for action potentials the impedance load of the soma (i.e., its contribution of conductance and capacitance) is relatively isolated from the T-junction. That said, for slower changes in membrane potential, the stem axon of unmyelinated fibers can be around 150 μm in length (Ha 1970) and therefore is likely to be electrotonically close to the T-junction.

**Fig. 3.**
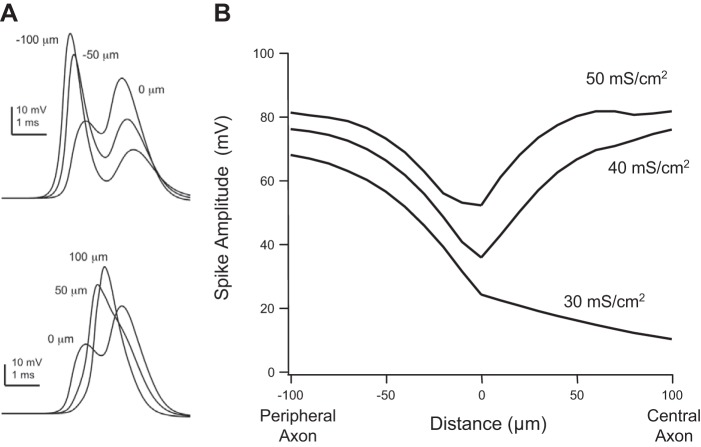
Action potential amplitude is reduced at the T-junction. *A*: voltage transients 100 and 50 μm in the peripheral axon before (*top* traces) and in the central axon after (*bottom* traces) the T-junction (distance = 0 μm). In the peripheral axon the waveforms exhibit a characteristic bimodal shape where the early mode is the orthodromic propagating spike and the later mode arises from the reflecting spike originating at the soma and stem axon. Note that in the central axon 50 μm from the T-junction, the waveform appears unimodal. *B*: spatial profile (±100 μm of the T-junction) of the early mode amplitude for 3 different values of voltage-gated Na^+^ (Na_V_) channel conductance density (*Ḡ*_Na_). At a density of 30 mS/cm^2^ the spike fails to actively propagate through the T-junction.

We then simulated the propagation of action potentials through the T-junction in a model containing fast voltage-gated Na_V_ and K_DR_ channels (see materials and methods). Spikes were monitored at varying locations ±100 μm on either side of the T-junction (Fig. 3*A*). A characteristic M-shaped bimodal waveform was observed proximal to the T-junction. It comprised an early, orthodromically propagating spike and a later spike reflected back from the soma. Within the central branch, distal from the T-junction at 50 μm, the waveform was dominated by the propagating spike. Under some circumstances, depending on the frequency of stimulation and variations in the properties of the model, the reflecting spike continued to propagate antidromically, toward the periphery (discussed further below), whereas it generally failed to invade the central axon due to the absolute refractory period of the propagating spike.

Plotting the early, orthodromically propagating spike as a function of distance either side of the T-junction illustrates the effect of the T-junction on action potential amplitude (Fig. 3*B*). Like with *Z*_N_, action potential amplitude decreased as it neared the T-junction and then regained amplitude as it progressed into the central axon. Spike amplitude and propagation were influenced by Na_V_ conductance density (*Ḡ*_Na_). In the peripheral axon, spike amplitude was generally proportional to *Ḡ*_Na_. In the central axon, spikes actively propagated through the T-junction when *Ḡ*_Na_ was 35 mS/cm^2^ or greater. At a critical value of *Ḡ*_Na_ (<35 mS/cm^2^), orthodromic spikes failed to cross the T-junction. Varying somatic *Ḡ*_Na_ had no effect on the spatial profile of spike amplitude and the reliability of spikes propagating through the T-junction (Amir and Devor 2003).

### DC Somatic Potentials Influence the T-Junction

**Fig. 4.**
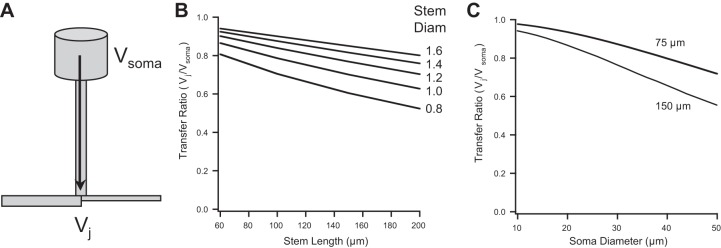
Voltage transfer from soma to T-junction. *A*: in a passive model the voltage transfer ratio was calculated from the steady-state voltage response at the T-junction (*V*_j_) relative to the steady-state potential originating at the soma (*V*_soma_). *B*: voltage transfer ratio plotted as function of stem axon length for a range of stem axon diameters. *C*: transfer ratio plotted as a function of soma diameter for 2 stem axon lengths.

Although somatic *Ḡ*_Na_ did not significantly affect propagation through the T-junction, experiments suggest that the steady-state or DC potential at the soma just prior to a spike invading the T-junction may affect propagation (Amir and Devor 1997; Du et al. 2014; Gemes et al. 2012; Hogan et al. 2008; Luscher et al. 1994a, 1994b). To better understand how DC signals at the soma might affect potential at the T-junction, we calculated steady-state voltage transfer ratio from the soma to the T-junction (*V*_j_/*V*_soma_) for varying stem axon geometries (Fig. 4*A*). Over a wide range of stem lengths (60–200 μm), steady-state voltage attenuation varied only 25% for an 0.8-μm-diameter stem axon compared with ∼5% for a 1.6-μm-diameter stem axon (Fig. 4*B*). When comparing models with a small-diameter soma to one with a larger diameter (10 vs. 50 μm), the difference in voltage attenuation ranged from ∼5% to ∼40% (for a model with a stem axon diameter of 1.4 μm and length of 150 μm). For comparison, for a shorter, 75-μm-long stem axon, the difference in attenuation ranged from ∼2% to ∼22% (Fig. 4*C*). This suggests that for a 10-mV steady-state hyperpolarization at the soma, the T-junction would be hyperpolarized by 6–8 mV. We would expect that DC voltage attenuation should be even less if the value for *R*_m_ were an underestimate, thereby making the stem axon electrotonically shorter.

### Low-Pass Filtering: Slow Outward Currents

**Fig. 5.**
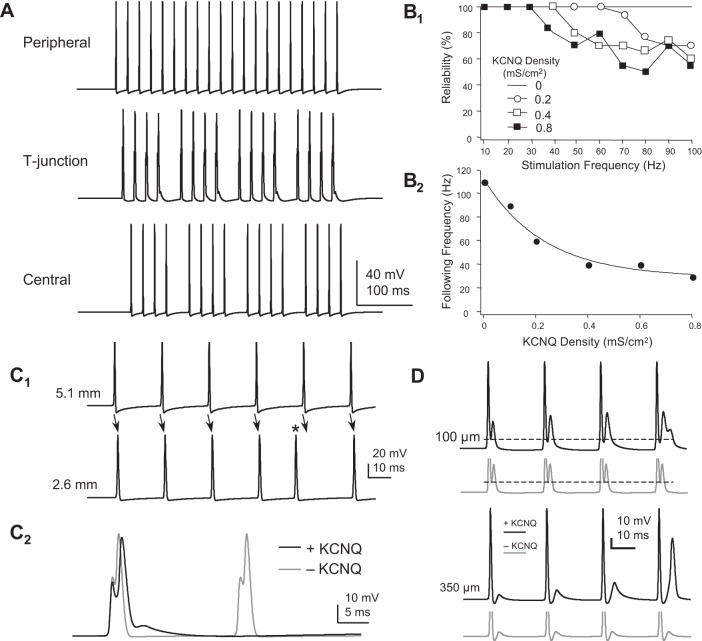
KCNQ density reduces following frequency through the T-junction. *A*: spike failure occurs at a stimulation frequency of 50 Hz in a model containing KCNQ channels (*Ḡ*_KCNQ_ = 0.4 mS/cm^2^, equivalent to 12 pA/pF at a *V*_1/2_ of −30 mV). Stem length = 75 μm. *B*: varying *Ḡ*_KCNQ_ affects following frequency. *B*_1_: the reliability of spike propagation (the percentage of spikes propagating through the T-junction) is plotted against stimulus frequency for a range of KCNQ densities. *B*_2_: following frequency plotted as a function of *Ḡ*_KCNQ_. Solid line is the best exponential fit. *C*: spike failure due to collision of orthodromic and antidromic spikes in the peripheral axon. *C*_1_: voltage-transients at 2 peripheral axon locations distal to the T-junction. At both locations there is reliable propagation of the first 4 spikes of a 40-Hz train from the most distal (5.1 mm) to a more proximal (2.6 mm) location from the T-junction. The fifth (ectopic) spike at the more proximal location (asterisk) appears before the fifth spike is evoked. This spike then fails to successfully propagate to the more proximal location. *C*_2_: waveforms corresponding to the fourth and fifth stimuli at the T-junction, with and without KCNQ channels. In the presence of KCNQ channels the fifth spike fails to propagate into the T-junction. *D*: the presence of KCNQ channels allows for the generation of an antidromically propagating ectopic spike. In the peripheral axon, 100 μm from the T-junction, with each of the first 4 successive spikes KCNQ channel activation augments the afterhyperpolarization (AHP) following each orthodromically propagating spike (dashed line represents the amplitude of the first AHP). The subsequent antidromic spikelet increases in amplitude. At a more distal peripheral location (350 μm from the T-junction), the antidromically propagating spikelet increases in amplitude with each successive pulse until it becomes suprathreshold for antidromic propagation on the fourth pulse when KCNQ channels are included in the model.

C-fiber sensory neurons fire at mean rates generally <20 Hz (Chen and Levine 2003; Leem et al. 1993; Long 1977; Yeomans et al. 1996; Yeomans and Proudfit 1996), although depending on the modality of the stimulus, instantaneous frequencies may approach 100 Hz (Chen and Levine 2003; Kress et al. 1992; Leem et al. 1993; Yeomans et al. 1996; Yeomans and Proudfit 1996). In the model containing only Na_V_ and K_DR_ conductances, propagation through the T-junction was highly reliable, up to a following frequency (the highest frequency where 100% of peripherally generated spikes propagated into the distal central axon) of 110 Hz (Fig. 5*B*_2_). Experiments demonstrate that spike propagation through the T-junction is generally quite reliable at frequencies slower than 5–10 Hz, whereas at higher frequencies spikes fail specifically at the T-junction (Gemes et al. 2012; Luscher et al. 1994b). A number of possible mechanisms might add to and influence the low-pass filtering properties at the T-junction of C-fibers. One possible low-pass filtering mechanism is the activation of KCNQ (K_V_7) channels expressed in the soma and axons of C-fiber neurons (Passmore et al. 2012; Rose et al. 2011). To determine if KCNQ channels might contribute to low-pass filtering, we added this slow voltage-gated K^+^ conductance to the soma, stem axon, and proximal (to the T-junction) peripheral and central axons. The presence of KCNQ channels in the model reduced the following frequency (Fig. 5*A*). With a KCNQ channel density (*Ḡ*_KCNQ_) of 0.2 mS/cm^2^ (corresponding to 6 pA/pF for currents at −30 mV), following frequency was reduced to 60 Hz (Fig. 5*B*). Increasing KCNQ channel density to 0.8 mS/cm^2^ (24 pA/pF) further reduced following frequency to 30 Hz. Increasing KCNQ density further had no additional effect on following frequency.

We expected that KCNQ currents might reduce conduction by producing a hyperpolarizing voltage shift at the T-junction and, in turn, decreasing the likelihood for spike propagation into the central axon. Trains of spikes did indeed trigger a KCNQ-dependent medium afterhyperpolarization of 2–3 mV (Fig. 5*A*), and the presence of KCNQ channels appeared to decrease the reliability of spike propagation through the T-junction. However, in the peripheral axon, at 2.6 mm from the T-junction, we noticed that an ectopic spike appeared following the fourth stimulus of a 40-Hz train (*Ḡ*_KCNQ_ = 0.4 mS/cm^2^). The fifth spike did not successfully propagate down the entire length of the peripheral axon. The fifth spike collided with the ectopic spike before it reached the T-junction (Fig. 5*C*_2_). Where did the ectopic spike arise and why do KCNQ channels promote antidromic spike generation? First, the action potential generated in the soma reflects back down the stem axon toward the T-junction. Normally, this spike fails to successfully propagate into either the peripheral or central axon due to *1*) the impedance mismatch, resulting in a smaller amplitude action potential, and *2*) the relative refractory period within the T-junction. In the peripheral axon, 100 μm before the T-junction, and with KCNQ channels, the fast AHP grew incrementally with each of the first four orthodromic spikes (Fig. 5*D*). In turn, the afterdepolarization produced by the somatic/stem axon action potential increased in amplitude due to Na^+^ channel de-inactivation. As a result, the fourth afterdepolarization, produced by the reflected spike generated at the soma, reaches threshold for antidromic propagation. Without KCNQ channels, the fast AHP and afterdepolarization amplitudes were not augmented in a frequency-dependent manner and antidromic spikes were not generated. The KCNQ channel in our model is based on a simple two-state model. KCNQ current activation may be more delayed due to multiple channel states (Lancaster and Pennefather 1987). Therefore, a single spike is not likely to sufficiently activate these channels. That said, low-frequency spikes (<40 Hz) produced very limited KCNQ channel activation and had no effect on antidromic spike generation. Only during a relatively higher frequency train of spikes (>40 Hz) was KCNQ conductance activated and antidromic spikes subsequently generated.

**Fig. 6.**
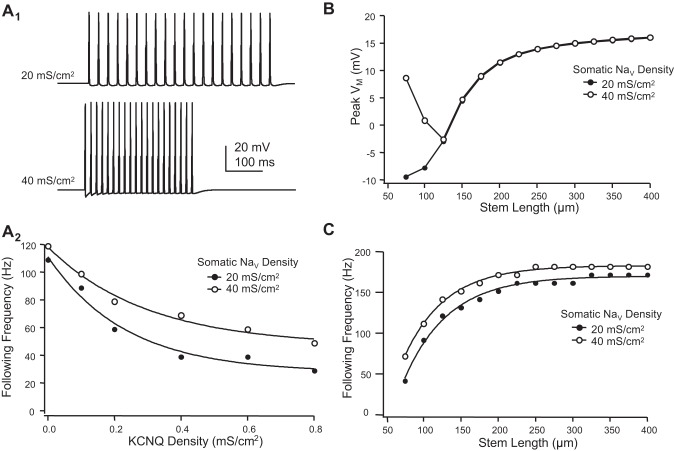
Interplay between Na^+^ channels and the M-current. *A*: effect of varying somatic Na^+^ (*Ḡ*_Na_) and KCNQ channel density (*Ḡ*_KCNQ_) on following frequency. Stem length = 75 μm. *A*_1_: following frequency was increased with greater somatic *Ḡ*_Na_. Spike trains (40 and 70 Hz) are shown for a model with *Ḡ*_KCNQ_ = 0.4 mS/cm^2^ and somatic *Ḡ*_Na_ of 20 and 40 mS/cm^2^. *A*_2_: following frequency plotted as a function of KCNQ and Na^+^ channel (Na_V_) density. *B*: action potential amplitude at the T-junction was affected by stem axon length. Varying somatic *Ḡ*_Na_ affected T-junction spike amplitude only for very short stem axons. *C*: electrotonically short stem axons influence following frequency. Stem axon lengths shorter than 200 μm decreased following frequency of spikes propagating through the T-junction. Varying the density of somatic Na^+^ channels had little effect.

The impact of KCNQ channels on following frequency could be countered by somatic Na_V_ conductance density. Following frequency through the T-junction was enhanced across a wide range of KCNQ densities by increasing the number of somatic Na_V_ channels (Fig. 6*A*). Doubling somatic Na_V_ channels increased following frequency from ∼35 to 50 Hz (Fig. 6*A*_2_). To determine the relative contribution of the soma's electrotonic distance and the influence of somatic Na_V_ channels, we monitored spike amplitude at the T-junction for varying stem lengths. When somatic *Ḡ*_Na_ was 50% lower than in the axons (the model's standard value), spike amplitude at the T-junction followed a sigmoid function of stem axon length (Fig. 6*B*). This was consistent with calculations of input impedance at the T-junction as a function of stem axon length (see Fig. 2*B*). When somatic *Ḡ*_Na_ was doubled, spike amplitude at the T-junction was boosted for short (<125 μm), but not long, stem axons (Fig. 6*B*). For both high and low somatic *Ḡ*_Na_, following frequency increased exponentially with stem length (Fig. 6*C*). These simulations emphasize that as the soma becomes electrotonically remote, its influence on low-pass filtering decreases.

**Fig. 7.**
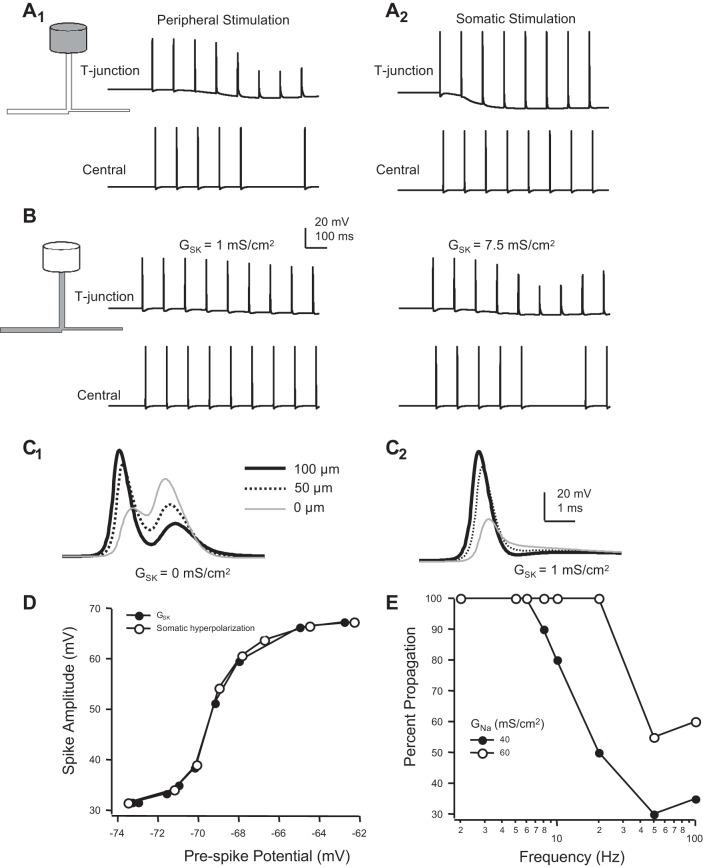
Slow hyperpolarizing voltage shift prevents spikes from propagating through the T-junction. *A*: somatic SK-like Ca^2+^-dependent K^+^ conductance (*Ḡ*_SK_ = 1 mS/cm^2^) produces a hyperpolarizing voltage shift that decreases the reliability of spike propagation (10 Hz) through the T-junction. In *A*_1_, spikes generated in the periphery fail to propagate into the central branch (5 mm from the T-junction) at the peak of the hyperpolarization. In the same mode, somatically generated spikes (*A*_2_) reliably propagate through the T-junction. *B*: SK-like channels expressed in the axons at a higher density effectively limit spike propagation. When SK channels were expressed in axons proximal to the T-junction at the same density as in *A*, spikes delivered at 10 Hz reliably propagated through the T-junction (*left* traces). When *Ḡ*_SK_ was increased to 7.5 mS/cm^2^, spike failure was observed (*right* traces). *C*: voltage transients 100 and 50 μm in the peripheral branch approaching the T-junction, and at the T-junction (0 μm). In the absence of SK channels (*C*_1_), bimodal voltage transient is observed at all 3 locations. When somatic SK channels are expressed, spikes that fail to propagate into the central axon are unimodal (*C*_2_). *D*: spike amplitude at the T-junction plotted a function of the shift in voltage, produced either by a somatic SK-like conductance or by somatic hyperpolarizing current injection. *E*: reliability of spike propagation through T-junction, plotted as the percentage of spikes reaching the distal central branch. At a *Ḡ*_Na_ of 40 mS/cm^2^, following frequency was 6 Hz. Increasing *Ḡ*_Na_ to 60 mS/cm^2^ increased following frequency to 20 Hz.

The kinetics of KCNQ channels alone do not appear to be sufficiently slow enough to filter spike trains at frequencies below 20 Hz (Gemes et al. 2012; Luscher et al. 1994b). We hypothesized that a spike-dependent conductance, even slower than that for KCNQ channels, producing a hyperpolarizing voltage shift at the T-junction (Amir and Devor 1997) might decrease the following frequency within this range. An SK-like Ca^2+^-dependent K^+^ current was chosen to test this hypothesis, although any slow, spike-dependent mechanism that achieves a relatively steady membrane hyperpolarization (e.g., K_Na_ channel activation) would be expected to have the same effect (see Fig. 7*D*). To the soma of the model we added *1*) a high-threshold voltage-gated Ca^2+^ current (Ca_V_), *2*) Ca^2+^ dynamics (i.e., accumulation, buffering, and extrusion/sequestration), and *3*) the SK-like current (K_SK_). At a critical SK conductance (*Ḡ*_SK_), action potential following frequency decreased to below 10 Hz (Fig. 7, *A* and *B*). In contrast, somatic stimulation reliably triggered spike propagation into the central axon (Fig. 7*A*_2_). Inserting Ca_V_ and K_SK_ channels (and intracellular Ca^2+^ dynamics) in axons proximal to the T-junction (but not the soma) was also effective at filtering signals. However, *Ḡ*_SK_ needed to be more than sevenfold greater. When SK channels were expressed solely in the soma, a density of 1 mS/cm^2^ was the minimum *Ḡ*_SK_ required to elicit failure at 10 Hz (Fig. 7*B*). When placed in the proximal axons (and not in the soma), the density required to elicit failure at 10 Hz was 7.5 mS/cm^2^. The hyperpolarizing voltage shift reduced spike amplitude at the T-junction and prevented somatic firing (Fig. 7, *C* and *D*). Amplitude of the orthodromic propagating spike at the T-junction was the primary determinant for spike propagation. Hyperpolarization prevented spike invasion into both the stem and central axon branches. This was consistent with the experimental observation that somatic spikes are not evoked when spike propagation fails at the T-junction (Gemes et al. 2012). Hyperpolarizing voltage shift at the T-junction, either by varying the maximum conductance (*Ḡ*_SK_) or by somatic hyperpolarizing current injection, had the same effect on the orthodromic propagating action potential amplitude at the soma, suggesting no significant effect of membrane conductance on spike amplitude (Fig. 7*D*). As previously described in Fig. 3, the influence of SK-like channels on spike propagation was most sensitive to variations in Na_V_ parameters. For example, varying *Ḡ*_Na_ produced significant changes in the reliability of spike propagation; increasing *Ḡ*_Na_ from 40 to 60 mS/cm^2^ shifted following frequency from 6 to 20 Hz (Fig. 7*E*).

**Fig. 8.**
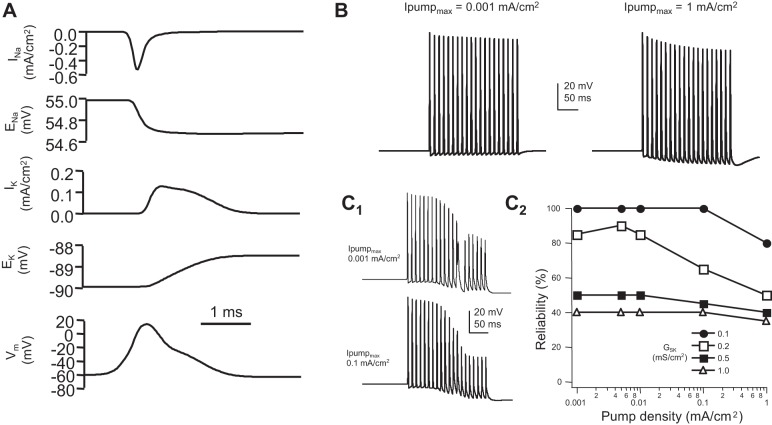
Influence of ion concentration and electrogenic Na^+^-K^+^ ATPase on spike propagation through the T-junction. *A*: Na^+^ and K^+^ currents (*I*_Na_ and *I*_K_) and changes to their equilibrium potentials (*E*_Na_ and *E*_K_) resulting from a single spike in the central axon 20 μm distal from the T-junction. At this time scale, and for a single spike, changes in equilibrium potential and the Na^+^-K^+^ pump have no noticeable effect on equilibrium potential or the spike waveform. *B*: effects of a low and high maximum Na^+^-K^+^ pump current density (*I*_pump_max__) on a 100-Hz train of spikes propagating through the T-junction. Note the decrease in spike amplitude (due to decreased driving force for Na^+^) and the hyperpolarizing voltage shift (due to the electrogenic Na^+^-K^+^ pump). *C*: the Na^+^-K^+^ ATPase contributes to a small effect on spike conduction when in combination with the voltage shift of the SK-like Ca^2+^-dependent K^+^ conductance. *C*_1_: effect of low (*top*) and high (*bottom*) *I*_pump_max__ on 100-Hz spike trains at the T-junction in a model containing the SK-conductance (*Ḡ*_SK_ = 0.2 mS/cm^2^). *C*_2_: reliability of spike propagation through the T-junction for a range of *I*_pump_max__ and *Ḡ*_SK_ values.

Two other slow mechanisms that might potentially contribute to spike failure are *1*) the loss of driving force due to the accumulation of intracellular and extracellular Na^+^ and K^+^ and *2*) hyperpolarization produced by the Na^+^-K^+^ ATPase. The effect of either of these would be augmented by the larger surface-to-volume ratio of the thinner central branch of the axon (Donnelly et al. 1998; Luscher et al. 1994a; Tigerholm et al. 2014). We examined the effects of varying *E*_K_, which would occur following the accumulation of extracellular K^+^. As expected, the effect of a loss of K^+^ driving force could be compensated by an increase in K^+^ channel conductance (not shown). We next simulated Na^+^ and K^+^ dynamics, assuming linear summation and no buffering (Frankenhaeuser and Hodgkin 1956; Hodgkin and Keynes 1956; Kushmerick and Podolsky 1969) and adding a Na^+^-K^+^ pump with a hyperbolic relationship to intracellular Na^+^ concentration (Canavier 1999). As shown in Fig. 8*A*, a single spike propagating into the central branch produced only a limited decrease in the driving force for both Na^+^ and K^+^. In a model containing a low density of Na^+^-K^+^ pumps, a high-frequency (100 Hz) train of 20 spikes produced only a modest decrease in spike amplitude and only a small depolarizing voltage shift of membrane potential (Fig. 8*B*). When the maximum pump current density was increased 1,000-fold, a more prominent decrease in spike amplitude and a strong hyperpolarizing shift were observed. The effect of K^+^ accumulation on membrane potential was overwhelmed by the electrogenic effect of the Na^+^-K^+^ pump. However, within this range of parameters, spike conduction was not affected. The influence of the Na^+^-K^+^ pump-influenced spike conduction was most pronounced when it was combined with the voltage shift produced by the SK-like Ca^2+^-dependent K^+^ conductance (Fig. 8*C*).

## DISCUSSION

The major findings of this study are that *1*) the T-junction will reduce the safety factor for spike propagation in an unmyelinated sensory neuron model based on peripheral and central axon morphology if the stem axon provides a sufficient conductance load, *2*) the geometrical properties of the T-junction alone cannot account for low-pass filtering below 100 Hz, and *3*) KCNQ channels can reduce following frequency down to ∼30 Hz, whereas *4*) a slower process, such as an SK Ca^2+^-dependent K^+^ conductance, is needed to limit firing at lower frequencies.

### Morphological Influences on Following Frequency

Filtering of propagating signals at an axon or dendrite bifurcation is a well-described result of an impedance mismatch between parent and daughter branches (Debanne 2004; Goldstein and Rall 1974; Zhou and Chiu 2001). For a propagating action potential, the impedance mismatch reduces the likelihood that it can traverse the bifurcation, and therefore, the safety factor for spike propagation is reduced. Substantial evidence from experiments on both myelinated and unmyelinated sensory neurons supports the hypothesis that the safety factor for spike propagation is indeed lower at the T-junction and may contribute to low-pass filtering of action potentials (Djouhri et al. 2001; Fang et al. 2005; Gemes et al. 2012; Luscher et al. 1994b; Stoney 1985, 1990; Zhou and Chiu 2001). Our model is generally consistent with experimental observations and previous models of unmyelinated sensory neurons (Du et al. 2014; Gemes et al. 2012; Luscher et al. 1994a, 1994b).

Within the DRG the difference between the diameter of the peripheral and central branches critically affects the ability of the T-junction to filter action potentials. The central axon of a typical C-fiber is roughly half that of the peripheral axon (Ha 1970; Hoheisel and Mense 1986; Suh et al. 1984; Zhang et al. 1998). This suggests that the “stepping down” of axonal diameter at the T-junction may be an evolutionary adaptation for tuning, if not preventing, low-pass filtering at T-junction. Like the model of Luscher et al. (1994b), the diameter of the stem axon needed to be as large as, or larger than, that of the peripheral axon to achieve a significant impedance mismatch (Fig. 1*B*_1_). This would not be needed if the central axon were the same diameter as the peripheral axon (Fig. 1*B*_2_). And in this case (i.e., if peripheral and central axon diameters are the same), the conduction velocity of orthodromic spikes should be equivalent to those of antidromic spikes.

The sudden decrement of axon diameter at the T-junction makes a strong prediction regarding CV and following frequency. Over a wide range of stem axon geometries, antidromic spike propagation (i.e., spikes triggered in the distal central axon) failed to propagate through the T-junction. Only when the density of voltage-gated Na^+^ channels was elevated to a critical level was antidromic propagation possible, but at the cost of raising the safety factor for orthodromic propagation. This conflicts with the properties of spike propagation through the T-junction of cultured or acutely isolated sensory neurons where spike filtering was equivalent for both orthodromic and antidromic spikes (Gemes et al. 2012; Luscher et al. 1994a). The computer model by Luscher et al. (1994b) also assumed that there was no difference in axonal diameter between the peripheral and central branches, although experimental measurements suggest otherwise (Ha 1970; Hoheisel and Mense 1986; Suh et al. 1984; Zhang et al. 1998). As such, they found that spike signaling was direction independent. Could the membrane properties between peripheral and central axonal membrane compensate for the drop in axonal diameter while at the same time preserving a low safety factor for orthodromic propagation? For example, if voltage-gated Na^+^ channel density was elevated in the peripheral axon, to permit more favorable antidromic propagation, the safety factor for orthodromic propagation across the T-junction was also raised. We found no combination or distribution of parameters that revealed such behavior. The example mentioned above reduced the ability of the T-junction to filter out high-frequency signals. Alternatively, it is possible that the difference between peripheral and central axon diameter may not be preserved in vitro. Indeed, the direction independence of CV reported by Luscher et al. (1994b) and Gemes et al. (2012) is consistent with this hypothesis.

If the soma of a sensory neuron is electrotonically close enough to the T-junction, it could possibly have significant influence over spike integration. For large-caliber sensory neurons, somatic excitability appears to have little functional consequence on spike propagation (Amir and Devor 2003), although there is evidence that variations in membrane potential immediately before an action potential invades the DRG can significantly influence spike propagation (Amir and Devor 1997; Hogan et al. 2008; Luscher et al. 1994b). For some small-diameter, unmyelinated nociceptors (C-fibers), the stem axon can be significantly shorter than for myelinated neurons, i.e., <200 μm (Ha 1970; Hoheisel and Mense 1986; Luscher et al. 1994b), and spike propagation in those particular neurons may be influenced by somatic membrane potential (Du et al. 2014) and by the modulation of somatic voltage-gated channels (see Fig. 6).

### Parameters Affecting Low-Pass Filtering

As discussed above, the T-junction represents a location where the safety factor for spike propagation is reduced. Action potential amplitude is reduced as it approaches the T-junction due to impedance mismatch. In the model containing only fast voltage-gated K^+^ and Na^+^ channels, spike conduction is only impaired at rates above 100 Hz. The relative refractory period provided by fast voltage-gated K^+^ channels and Na^+^ channels alone is comparatively short. To reduce following frequency to below 20 Hz, a slow, activity-dependent conductance was required in the model (Fig. 7).

Amir and Devor (1997) found that for most myelinated sensory neurons, progressive membrane hyperpolarization, most likely due to the activation of a Ca^2+^-activated outward current, occurs during the course of a 10- to 20-Hz spike train. However, sometimes a gradual depolarization, possibly due to “cross-excitation” between sensory neurons, was observed (Amir and Devor 1997, 2000). Gemes et al. (2012) reported that the AHP gradually decreased in amplitude with each spike during a train, while at the same time its time course broadened, rather than a progressive increase in the AHP with each spike. They therefore concluded that membrane hyperpolarization, and more specifically the AHP, was not the likely mechanism for spike failure since there was no correlation with the slow time course of membrane potential during a train. Instead, they suggested that an increase in membrane conductance, by the opening of gated ion channels, was a more likely explanation for spike failure. Our simulations, however, suggest that a shunting conductance, one without a voltage shift at the T-junction, although it is a contributing factor, is less likely to influence spike conduction on its own. Likewise, Luscher et al. (1994b) concluded that spike failure at the T-junction was due to not only increased membrane conductance but also Ca^2+^ channel inactivation and, to a lesser extent, a change in Na^+^ driving force. Furthermore, they also observed that hyperpolarization of the soma decreased the likelihood of spike failure, whereas depolarization had the opposite effect. One explanation for their former observation is that action potentials in developing sensory neurons or those in culture may have an ionic basis more strongly dependent on voltage-gated Ca^2+^ channels and may have different voltage-dependent dynamics than adult sensory neurons. In our model, the reliability of spike propagation across the T-junction was most influenced by membrane potential, and to a much lesser extent, membrane conductance and Na^+^ driving force.

Activity-dependent or extrinsic modulation of various membrane conductances within the DRG may therefore be potential regulators of spike propagation and provide for clinical approaches to limiting chronic pain (Krames 2014; Pope et al. 2013). For example, electrical stimulation of the DRG triggers analgesia in human patients and alleviates even most resilient types of neuropathic pain (Deer et al. 2012; Liem et al. 2013), possibly by decreasing the excitability of nociceptive neurons and increasing the likelihood that action potentials fail to propagate past the DRG (Koopmeiners et al. 2013; Lirk et al. 2008). We recently reported that extrinsic hyperpolarization of the DRG can reduce pain signals from reaching the spinal cord (Du et al. 2014). Enhancing Ca^2+^ influx in DRG reverses the peripheral excitability developed after nerve injury, presumably by enhancing Ca^2+^-dependent K^+^ channels (Hogan et al. 2008), an effect that further supports our conclusions.

Unmyelinated nociceptive neurons respond to mechanical and noxious stimuli at rates generally well below 100 Hz (Chen and Levine 2003; Kress et al. 1992; Leem et al. 1993; Long 1977; Slugg et al. 2000), whereas in some reports C-fibers reliably conduct responses at 2 Hz or below (Campero et al. 2004; Obreja et al. 2010). In both an acute in vitro DRG preparation and for cultured unmyelinated sensory neurons, following frequency through the DRG was generally, but not always, limited to 5–10 Hz (Gemes et al. 2012; Luscher et al. 1994a). Failure to traverse the T-junction at frequencies <100 Hz most likely requires a slower conductance mechanism, other than that provided by K_DR_ or other voltage-gated K^+^ channels with relatively fast kinetics. Slowly activating KCNQ channels are expressed in nociceptive C-fibers and could produce a hyperpolarizing shift in membrane potential in response to relatively low firing rates, potentially augmenting low-pass filtering (Du et al. 2014; Passmore et al. 2003; Waxman and Zamponi 2014; Zheng et al. 2013). Indeed, when incorporated into our model, KCNQ channels reduced following frequency to ∼30 Hz. However, even when extremely high densities of KCNQ channels were added, following frequency was never reduced to <30 Hz. The voltage-dependence of KCNQ channel kinetics limits their ability to hyperpolarize the membrane across prolonged interspike intervals. They simply deactivate too quickly at or below the resting potential (Delmas and Brown 2005). Nonetheless, pharmacologically augmenting KCNQ currents by retigabine (which produces a large leftward shift in the channels' voltage dependence) in the DRG decreases nociceptive signaling through the DRG (Du et al. 2014). Likewise, the analgesic effects of systemic administration of retigabine are also most likely mediated by modulation of M-channels in peripheral fibers (Hayashi et al. 2014).

In contrast, when a slower, SK-type Ca^2+^-dependent K^+^ conductance was added, the following frequency dropped to below 20 Hz, which is in good agreement with the literature (Gemes et al. 2012; Hogan et al. 2008; Luscher et al. 1994a). This is consistent with the experimental observation that activation of Ca^2+^-dependent K^+^ conductances or increasing Ca^2+^ influx decreases following frequency in an acute DRG preparation (Gemes et al. 2012). As noted and demonstrated by Luscher et al. 1994a, other mechanisms, such as the electrogenic effects of Na^+^-K^+^ ATPase activation, might equally produce the same effect. However, our simulations suggest that the voltage shift produced by Na^+^-K^+^ pumps alone may not be sufficient to affect spike propagation (Fig. 8). Although activation of the Na^+^-K^+^ pump produces a voltage shift, it does not affect membrane conductance. Additionally, a buildup of intracellular Na^+^ or extracellular K^+^ has been suggested as a mechanism to account for activity-dependent changes in spike conduction in C-fibers (Tigerholm et al. 2014). On its own, temporal summation of ion concentration does not appear to be substantial enough to affect spike propagation as well. In combination with another slow mechanism, a loss of driving force may be a contributing factor (Luscher et al. 1994a).

Our model also suggests that these channels do not necessarily have to be relegated solely to the soma to be effective. If slowly activating outward currents are expressed in the axons proximal to the T-junction at a physiologically reasonable density, they can produce effects similar to those of channels expressed solely in the soma, albeit at a higher density (Fig. 7).

The reliability of spike propagation across the T-junction was most sensitive to the properties of voltage-gated Na^+^ channels (see Figs. 3, 6, and 7). This is of particular interest because Na^+^ channels are upregulated following nerve injury (Waxman et al. 2000). Variations in Na^+^ channel density, activation, or inactivation can readily influence following frequency through the DRG. For example, the modeling of Choi and Waxman (2011) demonstrated how the differential expression of two types of Na^+^ channels may influence sensory neuron excitability. Likewise, a decrease in outward conductances would also be expected to raise the following frequency of spike transmission through the DRG, possibly also contributing to chronic pain sensation (Chien et al. 2007; Rose et al. 2011; Waxman and Zamponi 2014). Indeed, downregulation of multiple K^+^ channels (including KCNQ) is a general feature of many chronic pain conditions (reviewed in Du and Gamper 2013). Slow membrane mechanisms that influence membrane potential within the DRG are therefore potential therapeutic targets for the relief of chronic pain. For nociceptors, the DRG therefore represents an attractive therapeutic target for novel pain treatments (Deer et al. 2012; Koopmeiners et al. 2013; Krames 2014; Pope et al. 2013).

## GRANTS

N. Gamper was supported by Medical Research Council Grants MR/K021303/1 and G1002183.

## DISCLOSURES

No conflicts of interest, financial or otherwise, are declared by the authors.

## AUTHOR CONTRIBUTIONS

D.S. and D.B.J. analyzed data; D.S., N.G., and D.B.J. interpreted results of experiments; D.S. and D.B.J. prepared figures; D.S. and D.B.J. drafted manuscript; D.S., N.G., and D.B.J. edited and revised manuscript; D.S., N.G., and D.B.J. approved final version of manuscript; N.G. and D.B.J. conception and design of research.
